# Estimating 3D Leaf and Stem Shape of Nursery Paprika Plants by a Novel Multi-Camera Photography System

**DOI:** 10.3390/s16060874

**Published:** 2016-06-14

**Authors:** Yu Zhang, Poching Teng, Yo Shimizu, Fumiki Hosoi, Kenji Omasa

**Affiliations:** Graduate School of Agricultural and Life Sciences, The University of Tokyo, 1-1-1, Yayoi, Bunkyo-ku, Tokyo 113-8657, Japan; 0491342004@mail.ecc.u-tokyo.ac.jp (Y.Z.); 8015764991@mail.ecc.u-tokyo.ac.jp (P.T.); ayosh@mail.ecc.u-tokyo.ac.jp (Y.S.); ahosoi@mail.ecc.u-tokyo.ac.jp (F.H.)

**Keywords:** 3D modeling, leaf, stem, accuracy, MCP system, stereovision, MVS, SfM

## Abstract

For plant breeding and growth monitoring, accurate measurements of plant structure parameters are very crucial. We have, therefore, developed a high efficiency Multi-Camera Photography (MCP) system combining Multi-View Stereovision (MVS) with the Structure from Motion (SfM) algorithm. In this paper, we measured six variables of nursery paprika plants and investigated the accuracy of 3D models reconstructed from photos taken by four lens types at four different positions. The results demonstrated that error between the estimated and measured values was small, and the root-mean-square errors (RMSE) for leaf width/length and stem height/diameter were 1.65 mm (R^2^ = 0.98) and 0.57 mm (R^2^ = 0.99), respectively. The accuracies of the 3D model reconstruction of leaf and stem by a 28-mm lens at the first and third camera positions were the highest, and the number of reconstructed fine-scale 3D model shape surfaces of leaf and stem is the most. The results confirmed the practicability of our new method for the reconstruction of fine-scale plant model and accurate estimation of the plant parameters. They also displayed that our system is a good system for capturing high-resolution 3D images of nursery plants with high efficiency.

## 1. Introduction

For plant breeding and growth monitoring, accurate measurements of plant structure parameters and plant functioning are very crucial [1,2]. Over the past three decades, 2D imaging has been applied to a variety of aspects, ranging from structural analysis [3], stomata movements and photosynthetic diagnosis [4,5,6], herbicide control [7,8], disease detection [9] and yield estimation [10]. However, as 2D imaging is not robust against the occlusion of plant organs, such as overlapping leaves and stems and changing shapes during the measurement, most of these applications are either for large-scale analysis or for simple plant variables’ measurements at the early growth stage [11]. Recently, 3D imaging technologies for the robust measurement of plant breeding and growth parameters have emerged and been applied for solving this problem owing to the advance of sensing technology [2,12,13].

Among the applications of 3D imaging technology in agriculture, 3D model construction for plants [2,14] and properties’ estimation for plant growth [15,16] are the most noticeable. Today, there are many commonly-known data acquisition methods for these applications, including active and passive methods. Among the active sensing technologies, scanning LiDARs (Light Detection and Ranging) are usually used for constructing accurate and detailed 3D models, but such systems are usually expensive and complex [14,17]. In our previous research, we have evaluated the performance of portable scanning LiDAR for estimating the leaf inclination angle distribution, leaf area density profiles, vertical plant area density, forest structure, water storage, *etc.* [2,16,17]. However, scanning LiDAR has the shortcomings of not only high cost and much time consumed, but also different sensor properties and robustness against background illumination [18]. Moreover, as leaf shapes are difficult to capture accurately if the leaves move because of air movement, the scanning LiDAR measurements must be performed without any disturbance of air movement [19]. Apart from the above active technologies, depth-sensing cameras [20,21] are more easily deployable and a less complicated mode of 3D data acquisition, as they enable acquiring image data in real time about texture or lighting conditions. However, although the cameras are low cost and easily applied, the captured data are difficult to directly apply because of their low resolution and high noise.

Among the passive sensing technologies, Shape-from-X and stereovision are the most widespread methods of acquiring 3D data. They have been successfully applied in indoor and outdoor studies. For example, Omasa *et al.* [22] showed the 3D microscopic measurement of intact petunia seedlings for measuring shape and growth with a modified shape-from-focus method that incorporated a linear regression operator for 3D reconstruction. As for stereovision, it is also widely applied to reconstruct 3D models of plants [15] under indoor conditions. For example, some researchers [23] showed leaf wilt detection by stereovision. For outdoor imaging in a limited scope or at larger scales, there was also much work in the literature that introduced the accuracy of stereovision [24,25] and the classification of various plants with experimental variations of the environmental factors [26]. For example, this was applied for measuring outer leaf inclination angles of soybean plants and tracking their diurnal and nocturnal leaf movements [27]. Moreover, it was also used for guidance and navigation in the field [28]. In short, these applications were mostly conducted by using binocular stereovision. However, there are many limitations. Firstly, similar to 2D imaging technology, self-occlusion is still considered one of the limitations in stereovision in conducting various visual tasks, such as 3D scene reconstruction, target recognition, stereo matching, visual tracking, and so on [2,29]. Self-occlusion occurs when one part of an object is occluded by another part of the object from the camera’s view, which will affect the results of the above tasks. Besides, stereovision is easily affected by wind or strong sunlight in outdoor conditions. Stereo matching and accuracy vary with the kind of algorithm used. The local matching algorithm is truly efficient, but less accurate than the global one, which could be, computationally, very expensive [30]. Finally, its performance is poor for close-range observation of surfaces because of the homogeneous surface texture, which produces pockets of missing depth information of the plants.

In general, according to the comparison of the above active and passive sensing technologies under indoor or outdoor illumination conditions, we can find out that the best approaches for 3D reconstruction are often focused on a particular application and cannot be easily adapted for conducting complex measurements. For stereovision, it can be a promising approach if we can improve its self-occlusion and accuracy problems. Thus, in this study, we applied a Multi-View Stereovision (MVS) method to design a novel image acquisition system with several low-cost digital cameras under indoor illumination for 3D reconstruction.

The MVS method originated as a natural improvement of the two-view stereo algorithms. Instead of capturing two photographs from two different viewpoints, MVS would capture more viewpoints in between to increase robustness, e.g., to image noise or surface texture [31,32,33]. As only one viewing perspective makes modeling difficult and some areas might not have been covered sufficiently, the images captured from different and overlapping views can effectively reconstruct a scene. Harwin and Lucieer [34] also suggest data collection from different perspectives. However, for determining the intrinsic camera parameters and the position of the corresponding points from uncalibrated images taken by several cameras, other techniques to reconstruct both camera positions and 3D points are necessary. A novel solution is proposed to improve surface representation and construct 3D plant models through automated feature extraction and key point matching across images based on the Structure from Motion (SfM) algorithm. SfM is a stereovision technique of simultaneously estimating camera positions and orientations and 3D scene structure from an unordered image dataset [35]. This algorithm can mitigate the self-occlusion problem in the stereovision method. Moreover, compared to scanning LiDAR and other methods, the cost of producing SfM point clouds is very low. Thus, there is a great potential in applying the SfM algorithm to reconstruct the 3D model. In fact, the SfM algorithm and related techniques have been introduced and developed in the last two decades. For example, the Scale-Invariant Feature Transform (SIFT) operator [36] was introduced to provide a potent illustration of characteristics *in situ* and allows significant characteristics in other perspectives to be contrasted and matched. Bundle adjustment was applied to generate a cluster of 3D coordinates of matching characteristics. The SfM algorithm was originally applied to many ground-based applications. It was also applied widely in reconstructing cultural and urban features for 3D modeling [37,38,39]. Recently, there were some geographic applications based on aerial platforms for outdoor features [27,40]. However, the SfM method for plants has been demonstrated to be more difficult than for other characteristics due to their more complicated and irregular structures [41,42,43]. The SfM algorithm has also some limitations in dealing with missing data. In this study, therefore, we have studied the combination of SfM and MVS algorithms for optimizing photo consistency and auto-calibration with high accuracy.

According to our investigation, no studies have specifically reported 3D vegetation modeling and the accuracy obtained using the SfM and MVS algorithms in indoor conditions, especially for nursery plants [44,45,46,47]. Consequently, this study is necessary to develop a low cost and high accuracy Multi-Camera Photography (MCP) system to generate a 3D model for nursery plants based on the MVS and SfM algorithms. The novelty of our method lies in the integration of SfM and MVS algorithms in indoor conditions for nursery plants. According to our method, we designed our MCP-SfM image acquisition system and demonstrated its feasibility and effectiveness through many experiments. Our method has solved some problems or limitations of other methods, such as high cost, self-occlusion, low accuracy, information missing, noises, and so on. Therefore, our proposed approach is innovative. Our objective is to estimate and compare plant parameters with different cameras and lenses for 3D imaging. We will introduce some metrics for the qualitative evaluation of 3D imaging and its accuracy for different lenses used for 3D shape reconstruction under four different positions. We will finally discuss the accuracy discrimination of leaf and stems properties, as well as the characteristics and limitations of the proposed system.

## 2. Materials and Methods

### 2.1. Plant Material

Paprika seedlings were grown in a combined black four-square nursery box (35.5 cm length × 28.0 cm width × 7.6 cm high) with 9 small pots in an environmentally-controlled growth chamber for over 3 weeks after sowing. The pots were filled with artificial soil (mixture of vermiculite and perlite, 2:1, *v*/*v*). Plants were watered every two days with a 1:1000 dilution of HYPONeX nutrient solution (HYPONeX Japan Corp., Ltd., Osaka, Japan). They were illuminated for 12 h each day by LEDs and fluorescence lamps at a photosynthesis photon flux density (PPFD) of 50 mmol·m^−2^·s^−1^. The PPFD ratio of red and blue light was 4:1. The air temperature was 26.5 °C for 16 h in the day and 24 °C for 8 h at night. The relative humidity was 70% in the day and 90% at night. The intact nursery paprika plants were measured in the experiments.

### 2.2. Multi-Camera Photography-Structure from Motion System

According to the principle of MVS, the depth from images can be recovered by corresponding two or more perspectives of the same object. The result of this process is a 3D point cloud, in which each 3D point matches to a pixel in one of the photos. In previous studies [25,26,27], the binocular stereo method was usually applied to two photos captured with two cameras divided by a horizontal distance known as the “baseline”. In our system design, 4 cameras are used to compose the MCP-SfM system for obtaining the 3D point cloud data of nursery paprika plants’ structure. The distance between lens and plant is about 35 cm. The MCP-SfM system is able to obtain a high resolution by calibrating the cameras and images corresponding the 3D point coordinates and locations in actual measurement scales. At the beginning, AF (Auto Focus) mode was used. Then, MF (Manual Focus) mode was used for capturing the clear photos after adjusting the focus. The exposure settings are ISO100-6400 before capturing the image; we have calibrated each camera for lens distortion compensation. Along with the conditions, the camera settings can provide optimal results.

**Figure 1:**
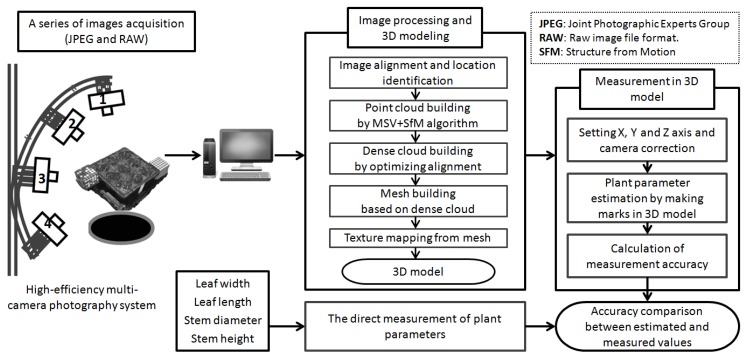
MCP-SfM system and the overall workflow for 3D modeling and measurement.

Figure 1 illustrates a schematic view of the combined MCP-SfM system. Four high resolution cameras (5184 × 3456 pixels; Canon EOS Kiss X7, Canon Industrial Co., Ltd., Tokyo, Japan) with 4 lenses of different focal lengths (24 mm, 28 mm, 35 mm, 50 mm) were used for the 3D range acquisition of individual plants. The camera positions (No. 1 to 4) on the adjustable bracket were fixed on the same side at four different angles. The left of Figure 1 shows the directions of the lens at each camera position. Positions 1 to 4 were 45° apart from each other in terms of vertical arrangement. In Position 3, the view direction of the camera was almost horizontal and can capture the entire object. The camera-to-leaf distance was between 35 and 40 cm. In order to obtain a high resolution view of the leaf and stem, the distance between the camera lens and the plants was slightly different for each camera position.

Image acquisition was done under indoor lighting conditions by varying the camera shutter speed. During the experiment, fluorescence lamps illuminate the entire plant in the four-square nursery box. The light adjustment exit was set so that the entire plant could be uniformly illuminated. In order to take the photos of the plant equably from a 360° view, a turntable and timer with a stepper motor were used. During the measurement, the monitor and remote automatic shutter were also used for the photography of all cameras. The images are taken at 5184 horizontal 3456 vertical pixels per frame with 8-bit resolution in the system by a Canon EOS Kiss X7 camera equipped with a polarization filter (Kenko Circular PL 58 mm for reflection removal, Kenko Tokina Japan Co., Ltd., Tokyo, Japan). These image datasets will be input into PhotoScan (Agisoft LLC, St. Petersburg, Russia) for calibration processing and 3D model building on the computer (see Figure 1). Besides, as the data collection and processing need more time, we used an automatic shutter and turntable for continuous acquisition of images to improve the photography acquisition efficiency. In this way, we were able to intermittently capture one photo per 3 s (rotation time) by controlling the automatic turntable. In total, about 8 min were needed for a rotation each time.

### 2.3. The MCP-SfM Approach

The MCP-SfM approach can be used to obtain 3D data of objects and to calibrate a cluster of camera positions and 3D point locations for each camera track [40,48,49]. In order to understand the approach well, we can conduct an incremental approach, estimating for a pair of images at a time, rather than matching all of the images for all of the cameras and tracks at once. The first step was to estimate the camera positions and 3D points for a single pair of images. The original pair should have many feature correspondences, but also a baseline, so that the 3D point locations of the observed views are in the optimum state. Then, 3D point coordinates and locations are calculated for the selected image observed with the most tracks. The next step is to add the tracks observed by the next cameras into the optimization. A track can be augmented if it is observed by at least one other camera and if triangulating the tracks offers an optimum estimation of their locations. This process is reiterated, one image at a time, until there are no remaining images to be perceived by any of the generated 3D points. To optimize the objective function at each repetition, we use the bundle adjustment algorithm [50], which was employed by using an SfM platform, namely Microsoft PhotoSynth.

**Figure 2:**
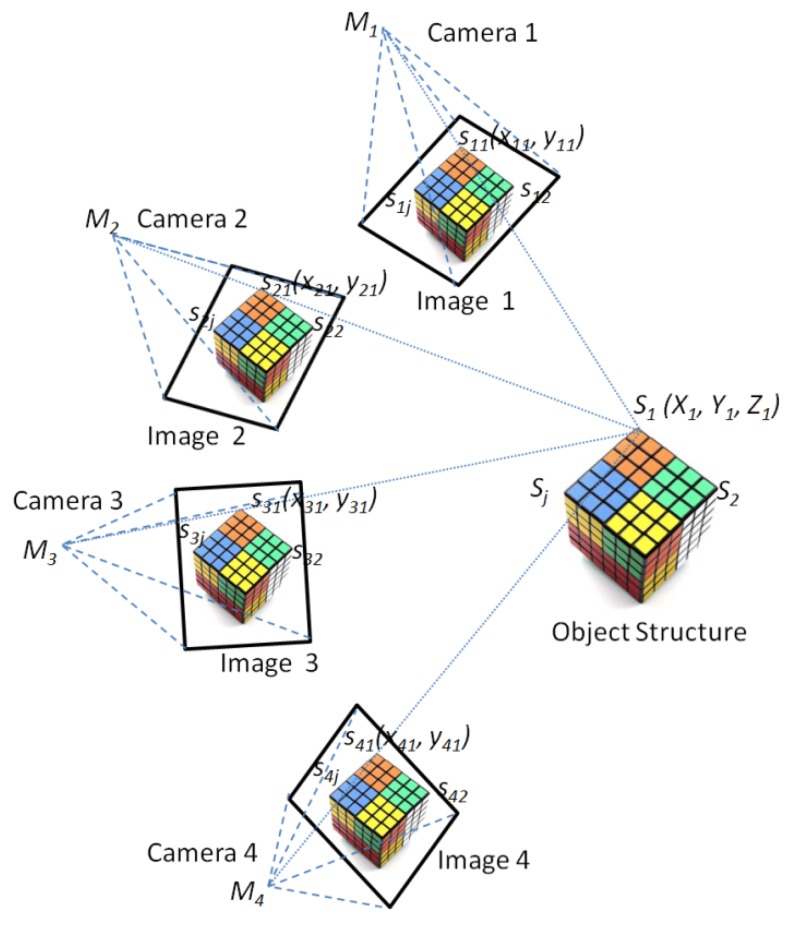
Methods for SfM for 3D model reconstruction.

The resulting SfM consists of an accordant arbitrary coordinate system, which should be georeferenced and converted to real coordinates. In lab conditions, SfM datasets consist of a set of normal referenced coordinate point data with additional color information from the photographs, such as a Rubik’s cube (see Figure 2). The key points generated from the SfM output can be located with *X*, *Y* and *Z* coordinates. Taking an example of using a pair of images by the SfM approach, the general process for solving the SfM problem is firstly to estimate the structure and motion up to a perspective transformation using the algebraic method or the factorization method proposed by Tomasi and Kanade [51]. For example, we need to estimate the *m* 2×4 projection matrices $M_{i}$ (motion) and the *n* 3D positions $S_{j}$ (structure) from the m×n 2D correspondences $s_{ij}$ (only allowing for translation and rotation between the cameras). This gives 2 m×n equations with 8m+3n unknowns that can be solved using the algebraic method or the factorization method.

(1) $$s_{ij}=M_{i}S_{j},\;i=1,...,\text{m};\;j=1,...,\text{n}$$

Some measurements are computed with extractive points from the SfM datasets that are matched to parameter estimations (number of points, point heights, *etc.*). Then, we can convert from perspectives to metrics by auto-calibration and minimize reprojection errors by optimizing the location of 3D points and the camera by applying bundle adjustment.

Similarly, as for the MCP-SfM approach with multiple images (see Figure 2), we need to find a common scale so that multiple images can be combined. One method is to use the Perspective N-Point (PnP) algorithm [52], where we try to solve for the position estimation of a new camera using the scene points we have already found. Typical SfM methods can robustly correspond characteristics in as many pairs of input photos as possible, so as to recover the estimations of the relatively accurate positions between photo pairs. Then, the bundle adjustment [53] calculates a maximum likelihood estimation of the camera positions and point positions, after initialization by a subset of the coupled estimations.

In this method, instead of increasing a single camera into the optimization, we increased several cameras at a time. As for selecting which cameras to increase, we originally need to get the camera with the most correspondence characteristics, *C*, to the subsistent 3D point clouds, then increase any camera with at least 75% *C* correspondences to the subsistent 3D point clouds. Generally, key point correspondence is very difficult when working with plant characteristics due to leaf sizes and gaps, repeating and inconsonant geometric structures, *etc.* [53]. Accordingly, some parts with few surface features may not be well captured; similarly, other parts with complicated geometric structures may not be well mapped or appear with radial distortion. Therefore, we implement the SfM algorithm to generate more precise point clouds and surface models at greater detail in spatial applications than the previous methods.

### 2.4. 3D Model Processing of Nursery Plants

**Figure 3:**
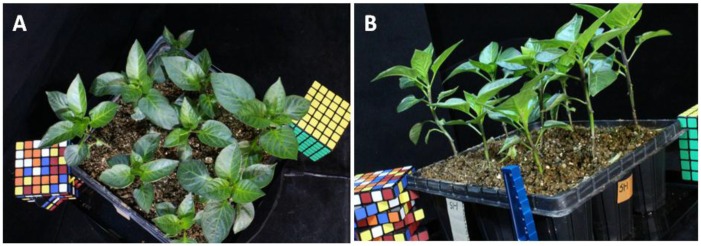
Original photos of paprika plants taken by the MCP-SfM system. (**A**) One of the original photos taken by a 28-mm lens at the 1st camera position; (**B**) One of the original photos taken by a 28-mm lens at the 3rd camera position.

Figure 3 shows the original photos of paprika plants taken by the MCP-SfM system. In Figure 3A, one of the original photos was taken by a 28-mm lens at the 1st camera position. The paprika canopy and leaf had a very high resolution. In Figure 3B, one of the original photos was taken by a 28-mm lens at the 3rd camera position. The paprika stem shows a very high resolution, as well as the references.

Based on the MCP-SfM approach, the uncalibrated original image data were processed and calculated by PhotoScan. In summary, there are 5 steps of image processing during the 3D model rebuilding, as shown in the middle of Figure 1. As we know, the data taken from 4 different measuring views had individual coordinate systems. First of all, in order to make 4 cameras have the same 3D coordinate system, the image datasets obtained from 4 camera positions were co-registered into the orthogonal coordinates and aligned by location identification.

Because of the estimated matching points and camera positions, the datasets are registered through an accurate conversion. Then, the conversion was repeatedly extracted by selecting in turn matching points from the image dataset and getting the best translation and rotation matrices that minimize the error estimation on account of the distance between them. This process was conducted for all couples of the image dataset. Then, the colored point clouds of the stereo image data captured were produced with 4 calibrated stereo cameras by the MSV and SfM algorithms after calibrating the stereo camera system. These points are often located by *X*, *Y* and *Z* coordinates in the 3D coordinate system. The MSV algorithm starts from the calibrated stereo images and a set of tracks. For properly-calibrated stereo cameras, the non-distortion of stereo images is actually combined with rectification. The process computed disparity images from incoming stereo images using the block matching algorithm. The disparity images were generated using a local correlation algorithm based on Sum of Squared Differences (SSD). It was optimized for efficiency and has a number of validation steps to prune disparity. Note that the extracted 3D point clouds were entangled with redundancies, a large number of outliers and noise. Fortunately, the additional information provided by the calibrated images can be exploited to help mesh and surface reconstruction later. After exporting 3D point data, the dense point cloud model was reconstructed in four steps, which includes calculating the depth information for each camera, capturing dense point cloud correspondences and reconstructing the structure from motion and dense 3D point clouds. The dense point clouds could be edited and classified or exported to an external tool for further analysis. Then, the classified dense point clouds of the plant were transformed into polygon meshes. This procedure did some basic filtering to eliminate the most obviously irrelevant points. Thereafter, such polygon wireframe images of the plant allowed calculating the distance or area of leaves and stems.

Therefore, the 3D surface texture of leaves and stems was expressed, as the inconsistent triangle meshes depend especially on the alignment of the point cloud after noise exclusion. Moreover, triangulated meshing and surface reconstruction were applied to generate surface texture models. Laplacian smoothing was employed to smooth triangle meshing. A series of natural color photos captured after wireframing were matched on the surface model of the entire plant using a texture-mapping approach. The texture-mapping approach is generally used as a method for putting surface texture into a computer-reconstructed 3D surface model [54]. The 2D photos like natural color photos are available for the surface model reconstruction. Through the coordinate’s correspondence of the 2D image to the 3D model, the feature of the 2D image is automatically matched onto each wireframe shell of the 3D model. A rigid choice of the matching points between 2D and 3D images is very important to generate the complicated texture mapping of the paprika. Then, the matching points were selected from unusual points on each stem and leaf; whereafter, exact 3D natural color models were constructed. From the models, some information can be refined, such as plant diameter and height, leaf shapes and area, which are significant in plant detection and recognition.

### 2.5. 3D Model Measurement of Nursery Plants

In order to estimate the parameters of the plant from the 3D model, we choose the two clearly-distinguished Rubik’s cubes on two sides of the nursery plant box as references to set the referenced coordinates’ points because of their standard 3D structure. The length of each edge has a fixed value of 66 mm. The coordinates in one of the two cubes were set as reference coordinates with three-dimensional coordinates (*X*, *Y*, *Z*). These georeferenced point clouds can be used to provide very specific and exact representations of a close depth scene. The measurement parameters include leaf width, leaf length, stem diameter and stem height. According to the coordinates of the referenced points in the 3D model, they were estimated by calculating the distances of the measured marked points in each leaf of the 3D model. For the measurement of each leaf, the distance between the upper end of the petiole to the leaf apex was calculated as the leaf length along the leaf vein. The distance from one edge to another edge in the middle part of the blade was calculated as the leaf width. As for the measurement of the stem, the stem diameter was calculated in the center of the stem. Stem height was calculated from stem tip to soil surface. In this experiment, 18 leaves and 9 stems were chosen from the 3D model and about 100 points on the 3D model were marked for the measurement in each leaf and stem, as well as the cube reference. For the direct measurement of plant growth parameters, we adopted the destructive method to measure the leaf, because plant leaves have irregular shapes and different states, such as wide or narrow, straight or curly, *etc.* In the experiment sample pot, there were 9 paprika plants in total. We selected two sample leaves from each paprika plant. We cut and measured them by a ruler. The measurement method for every leaf and stem is the same as the above. Finally, from leaf width and length to stem diameter and height, including spatial summaries of nursery plants, we could obtain the direct and indirect measurement data. According to these data, we could calculate correlation coefficients and Root-Mean-Square Errors (RMSE) between the estimated and measured values of these parameters to obtain their accuracy. We were also able to obtain the percentage of the total number of virtual leaves and stems whose 3D model surface area reconstructed over 95% or 75%, accounting for the total number of real leaves and stems, to compare the performance of different lenses.

## 3. Results

### 3.1. 3D Modeling Reconstruction of Paprika Plant

Based on the MSV and SfM algorithms, we have applied the high efficiency MCP-SfM system to obtain many high resolution images from close-up views of four positions. We have respectively tested four types of lenses for one circle of plant shape in four different camera positions, whose focal lengths were 24 mm, 28 mm, 35 mm and 50 mm. Table 1 shows that the numbers of acquired images of different lenses in the first and second camera positions, which were on average about 42 photos. However, for the third and fourth camera positions, the number of acquired images of the 50-mm lens is the smallest, because this lens is more sensitive to light intensity in the third and fourth camera positions. Because the MCP-SfM system has been set with certain shooting distance and the indoor light condition, Table 1 demonstrates that the 28-mm lens had good performance while taking photos of nursery plants.

**Figure 4:**
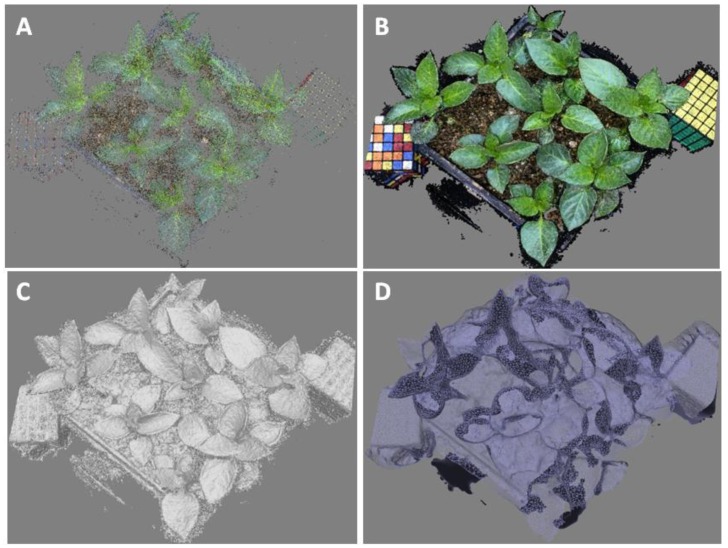
Procedure of 3D modeling for paprika plants reconstructed from a series of photos taken by the 28-mm lens at the first camera position. (**A**) Point cloud; (**B**) Dense cloud; (**C**) Dense cloud classes; (**D**) Wireframe.

In terms of the resolution and performance of different lenses, we found that 28 mm was better than other focal lengths. The features of the paprika SfM model are shown in Figure 4.

Figure 4 showed the procedure of 3D modeling for nursery paprika plants reconstructed from a series of photos taken by the 28-mm lens at the first camera position. Firstly, according to the process of 3D modeling, PhotoScan generated point clouds with a total of 88,022 points after registration from the 44 input images captured by the camera, as shown in Figure 4A. The number of points per leaf ranged from 105 to 5140, depending on leaf size. Currently, each point in the clouds of points is determined by two or more images, and overlapping areas have a lot of points. It would be more accurate if this were determined by all possible images, with fewer points, but more precision in the points. It is best to define the minimum number of images for the determination of a point. Secondly, after removing the noise within the point cloud dataset, a total of 1,528,730 dense points remained in Figure 4B, of which 1,528,730 points were classified as shown in gray in Figure 4C. The dense point clouds gave greater precision, and the point number is thirty times more than one of the point clouds and much more accurate. Then, the classified dense clouds of plant leaf were converted into the corresponding polygon wireframe in Figure 4D. It was seen that the polygons consist of many inconsistent triangle wireframes. The complex curves and unevenness on the paprika plant model were generated on the polygon wireframes. The number of polygon wireframes per leaf ranged from 139 to 5765, which was determined by leaf size.

Typically, while building the point clouds, noise also increased with increasing point density. The noise points were a little higher or lower than that expected, which cannot reproduce any actual characteristics within the paprika plants. Besides, the extent of the point cloud dataset was also decreased to minimize processing time, and the point clouds outside of the region were directly classified as noise points. Most of these were removed during building the dense clouds. The filtered point clouds produced an accurate 3D modeling of the paprika plants, as shown in Figure 4. Each leaf or stem feature was clear because of the high-resolution photos captured by the system.

**Figure 5:**
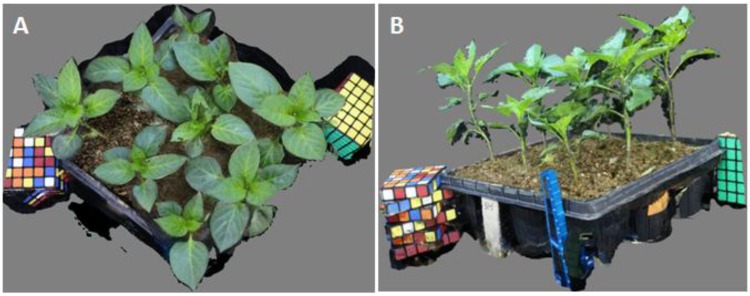
Texture mapping of the best 3D models for paprika plants. (**A**) Leaf models reconstructed from a series of photos taken by a 28-mm lens at the first camera position; (**B**) Leaf and stem models reconstructed from a series of photos taken by a 28-mm lens at the third camera position.

Figure 5 shows the texture mapping of the best 3D models with 0.25-mm resolution for paprika plants by a 28-mm lens in the first and third camera position. In Figure 5A, leaf models are reconstructed from a series of photos taken by a 28-mm lens at the first camera position. In Figure 5B, leaf and stem models were reconstructed from a series of photos taken by a 28-mm lens at the third camera position. Comparing Figure 5A to Figure 5B, the first camera position had a higher resolution. Moreover, Figure 5 also shows that paprika models in outer pots are generally better than ones in inner pots by the 28-mm lens in the details. Furthermore, even lower parts of nursery plant shapes can also be estimated and reconstructed. In sum, from Figure 5, we can see that most of the leaf and stem shapes were reconstructed, and their clear models were generated by the 28-mm lens in the first and third camera positions, respectively.

### 3.2. Parameters’ Estimation of the 3D Paprika Plant Model

According to the set georeferenced coordinate system and created measurement points in the 3D model, the estimation for leaf and stem shape parameters was conducted. For example, in Figure 5A, the generated leaf lengths ranged between 31 mm and 64 mm over all paprika plants. Likewise, in Figure 5B, the generated stem heights ranged between 53 mm and 96 mm. The difference of nine paprika stems’ height between the measured height and the estimated height was found to be less than 1.14 mm. The difference of nine paprika stems’ diameter between the measured value and the estimated value was under 0.76 mm. The average difference of stem height and diameter was about 0.6 mm and 0.31 mm, respectively. Figure 5 demonstrates that the 3D model reconstructed by the 28-mm lens in the first and third camera positions is better than other lenses in other camera positions and camera position groups.

Table 2 illustrates the percentage of the total number of virtual leaves and stems whose 3D model shape surface area reconstructed over 95% (Category A) or 75% (Category B) accounting for the total number of real leaves and stems. Ninety five percent and 75% mean the percentage of the reconstructed surface area of each leaf and stem shape compared to the real shape in the 3D model. Category A means the percentage of the 3D model shape surface area reconstructed over 95% compared to the real shape for both leaves and stems. Category B means the percentage of the 3D model shape surface area reconstructed over 75% compared to the real shape for both leaves and stems. The values in Table 2 were obtained by calculating the total number of virtual leaves and stems whose 3D model shape surface area reconstructed over 95% or 75% divided by the total number of real leaves and stems in the pot. For example, the total number of real plant leaves is 74. For the 28-mm lens in the first camera position, the total number of virtual leaves whose 3D leaf model shape surface area reconstructed over 95% is 35. Therefore, by dividing 35 by 74, the approximate value of 0.4729 can be obtained. Hence, the percentage of the total number of virtual leaves whose 3D model shape surface area reconstructed over 95% accounting for the total number of real leaves is about 47%, as seen in Figure 5A. Other values for leaves in Table 2 were obtained in the same way. In other camera positions or camera position groups, the percentage of the total number of virtual leaves whose 3D model shape surface area reconstructed over 95% accounting for the total number of real leaves is mostly less than 40%. Likewise, the values for stems in Table 2 can also be obtained in the same way. As can be seen in Table 2, for the 28-mm lens in the third camera position, the percentage of the total number of virtual stems whose 3D model shape surface area reconstructed over 95% accounting for the total number of real leaves is about 100%. Therefore, all of the stem shapes were reconstructed by the 28-mm lens in the third camera position, as seen in Figure 5B. However, in other camera positions or camera position groups, the percentage of the total number of virtual leaves whose 3D model shape surface area reconstructed over 95% accounting for the total number of real leaves is mostly less than 90%. Thus, from Table 2, we can see that the 28-mm lens in the first and third camera position has great performance in reconstructing the 3D model of the plant.

In general, according to the parameters’ estimation result of the 3D paprika plant model, we can analyze the performance of different lens types in different camera positions. These analyses are very helpful for understanding the monitoring of plant growth parameters and the environment, such as light conditions and disease detection.

### 3.3. Error Measurement of the 3D Paprika Plant Model

In our experiment, the error analyses of the estimated parameters, such as the correlation coefficient and RMSE, were conducted for different lens types and camera positions. According to the error analyses’ comparison, the results of the 28-mm lens were demonstrated to be the best. Some models generated by other lenses are not so good, with more noise and calibration errors in some parts of the 3D model. For example, the absolute error of the No. 1 paprika stem height for nine paprika stems is 0.792 mm. Its relative error is 0.008, which is obtained from 0.792 divided by 99 (measured value). The absolute error of height for nine paprika stems is under 0.2 mm.

**Figure 6:**
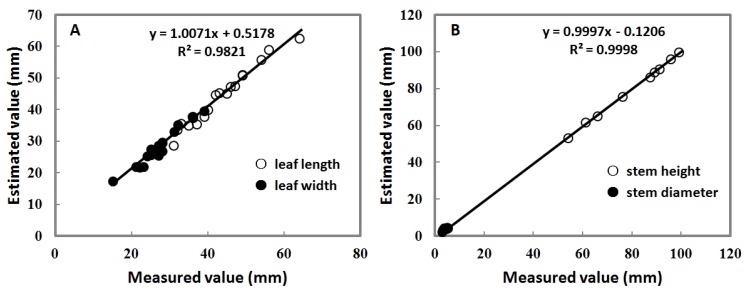
The accuracy of the best 3D models for leaf and stem shapes of paprika plants taken by the 28-mm lens. (**A**) Leaf length and width of plants taken at the first camera position; (**B**) Stem heights and diameters of plants taken at the third camera position.

Figure 6 shows the coefficient of determination (R^2^) and the regression results of leaf and stem for the 28-mm lens. The measured and estimated values were showed in a one-to-one relationship and a regression fit trend line. This demonstrated the accuracy of the best 3D models for leaf and stem shapes of paprika plants taken by the 28-mm lens. In Figure 6A, for the leaf length and width of plants taken at the first camera position, the correlation coefficient between the measured and estimated values showed a good fit. Likewise, in Figure 6B, for stem heights and the diameters of plants taken at the third camera position, the regression fit between the measured and estimated values was higher, giving an R^2^ of approximately 1.0. Besides, the spatial resolution was 0.25 mm per pixel on the *Y*-axis and 0.329 mm per pixel on the *Z*-axis. The accuracy assessment is high with a mean difference of the *Z*-values of 0.6 mm. Therefore, the accuracy of the 3D model by our MCP system is reliable.

In addition, the RSME of the errors was computed along with the regression fit degree and R^2^ between measured and estimated parameters. As can be seen in Table 3, the error results of the 3D model of best leaf and stem taken by the 28-mm lens are illustrated. The R^2^ and RMSE of paprika leaf and stem were presented for the whole experiment according to the measurement technique and method. In the first camera position, it showed a correlation coefficient of 0.9821 (*n* = 18) between the measured and estimated values with a mean difference of 1.65 mm. Likewise, in the third camera position, the RMSE of stem was 1.65 mm, and it showed a correlation coefficient of 0.9998 (*n* = 9) between the measured and estimated values with a mean difference of 0.57 mm. Besides, Table 3 shows better accuracy of estimation for stem than for leaf in the case of the 28-mm lens. The estimation difference between paprika stem and leaf was because of the limitations of the remote sensing method.

All in all, the above results demonstrated a general consistency between the estimated and measured values for 3D plant model reconstruction by analyzing the statistically-significant differences among different lens types, pointing out the promising role of the MCP system with several consumer-grade cameras for the purposes of plant growth monitoring.

## 4. Discussions and Conclusions

### 4.1. Advantages of the MCP System Based on the MVS and SfM Methods

This study presented an evaluation of the accuracy of the 3D image model derived by the MCP-SfM system based on the MVS and SfM technologies. The techniques are usually based on the consistency measurement, where the 3D model is consistent with the input images. They provide viable methods for generating 3D models of vegetation and scenes. The MVS method is a common application of 3D modeling of nursery plants. The SfM method is used to generate 3D models for triangulated meshing and surface reconstruction. These models generated by different lenses or camera positions were compared and evaluated. In general, the SfM method has a great potential in remote sensing range data capture of natural and man-made landscapes to provide very detailed and precise representations. Some small experiments indoors, like the one presented in this study, can be easily monitored using the system. Producing multi-temporal datasets of the entire growth period is essential for obtaining reliable results in such experiments. In the present study, we measured the performance of the highly effective and low cost systems with digital cameras and image reconstruction techniques for estimating the four variables of paprika plants, such as leaf width and length and stem height and diameter. The results showed the practicability of our new method for an exact measurement of plant parameters. The system can make the construction and acquisition of images faster and generate surface models from more viewing angles. The system is also available for extension into real-time monitoring of plant growth in the future. Therefore, it is an ideal system platform for taking more high resolution 3D images of plants.

### 4.2. Error Assessment of the 3D Paprika Plant Model

For the MCP-SfM system, the challenge is the performance and accuracy of the SfM algorithm and the accuracy of 3D depth data with the effect of ambient illumination. In fact, apart from the error analysis of the 3D paprika plant model, the accuracy of depth data is usually divided between the absolute and relative errors. The absolute error is obtained by the comparison with a known reference value. In the presented case, the absolute error is obtained by comparing the estimated value to a measured one. The relative error can be estimated by calculating the absolute error (the difference between the estimated value and the measured value) divided by the measured value. It gives an indication of how good a measurement is relative to the size of the object being measured. Therefore, the comparative accuracy of these measurements can be determined by looking at their relative errors. Besides, the relative error in the parameters’ measurement can better reflect the reliability of measurement accuracy. In particular, this could be considered as one of the reliable evaluation tools in future Digital Elevation Model (DEM) studies.

The precisions achieved on the individual paprika displayed variations determined by the type of the lens and the position of the camera. According to our various experimental comparisons, a focal length larger than 35 mm could not better reconstruct high resolution 3D models because the measurement devices were installed in a limited scope for indoor conditions. Besides, the upper camera positions were better for the measurement purposes of leaf shape in the 3D model, whereas the lower camera positions are better for the measurement purposes of stem shape. The camera position groups were better for the measurement purposes of the whole plant shape in the 3D model. In general, the performance was the best using the 28-mm lens in the first and third camera positions. According to Table 3, the RMSE result of these errors was considered an acceptable error range for estimating the features in consideration of the possible errors’ magnitude during manual measurements of the parameters, which could give the validity of the reference data [55,56]. Errors were slightly higher for other lenses or camera positions. What is more, in the above studies, we also found low correlations between the estimated and measured variables, which showed the influence of some limitations of this condition.

In addition, according to the percentage of the total number of virtual leaves and stems whose 3D model shape surface area reconstructed over 95% or 75% accounting for the total number of real leaves and stems (Table 2), the errors for the reconstructed 3D model are obvious among different focal lengths and camera positions. The main reasons for the errors in the parameters’ measurement might be not only different overlap rates due to different captured image numbers, but also different exposure rates due to some particularities of the ambient illumination condition. Another reason is the distance between paprika plants and the camera lens in the indoor experimental site, because some larger focal lengths need a longer distance, such as 50 mm and 85 mm. Thus, it is important for higher 3D model accuracy to adjust the suitable ambient illumination condition and shooting distance in the limited lab scope based on the lighting conditions of different camera positions and the characteristics of different lenses.

### 4.3. Limitations and Issues of the Experiment

In the construction of point clouds, the noises faced in some areas with a complex structure result in sparse black patches, which need to be well represented. According to our investigation, there are some limitations of the imaging system combined with environmental factors. One of the most important factors is light. Generally, lighting should be diffused to reduce errors. Under indoor conditions, the background and shading arrangements have been used to cater to the experiments. In this way, the effect of ambient illumination for the camera varies with the type of lens used. As the quantity, resolution and luminosity of images captured from the cameras of different views are different, there are some differences among different 3D models. In this study, we have discussed the exposure of the camera for the 3D imaging system, which is robust to variation in ambient illumination. It is necessary to compare and discuss the effect of lighting variations under different conditions on the plant canopies in the future. Besides, according to the different quantity of images and the performance of different computers, the entire workflow for producing 3D models from MCP-SfM images requires different time. For stereo images with dimensions of 5184 × 3456, it is important to arrange a uniform-luminance environment and a high-performance computer. Keeping the above limitations aside, it is also necessary for further research in the close range stereo imaging of plants to particularly take into account color distortion due to the lighting, sensors and exposure control, as discussed by Nielsen *et al.* [57]. Moreover, there are inherent limitations of the stereo matching process in this system, which is not robust to all sorts of surfaces and objects expected in agricultural scenarios. This reduces the effect of stereovision and limits either the range or the scale of the application. Finally, other issues with the system are the high performance requirement for the CPU and the large memory needed, especially when we try to do 3D reconstruction at a large scale.

### 4.4. Future Improvements and Applications

According to the above-mentioned issues, some improvements should be made in the future. In order to ensure that the camera in nadir position can take high quality images while capturing images, an adjustable light needs to be fixed for increased resolution. Additionally, we will further propose a method for obtaining the most suitable camera configurations for 3D imaging under different illumination conditions based on observing the precision trends in the camera and lens. Besides, an electrical trigger will also be used to make image acquisition more reliable compared to a mechanical shutter. Meanwhile, taking other sources of error into account, like the inaccuracies caused by rotating plants during data acquisition, the overlap of 90% among the images will be increased for higher accuracy in the future. Different settings were tested in PhotoScan showing that the model quality increased with the number of photos used for model generation. This is also stated by Roberts *et al.* [58]. Thus, in order to enable plant growth monitoring with higher accuracy, the number of photos taken during the illumination will be increased with a greater variety of viewing perspectives in the future. According to our next design, six more cameras will be installed in the system. Moreover, in the future, the models will also be expected to detect the differences between growths according to the cultivar and treatment.

## Figures and Tables

**Table 1:** The number of images taken by different lenses.

Camera Positions	Focal Length of Lens			
	24 mm	28 mm	35 mm	50 mm
1	44	44	44	40
2	42	41	44	42
3	42	43	42	29
4	42	45	43	23
Total	170	173	173	134

**Table 2:** Percentage of the total number of virtual leaves and stems whose 3D model shape surface area reconstructed over 95% (Category A) or 75% (Category B) accounting for the total number of real leaves and stems.

Camera Positions (see Figure 1)	Percentage of the Number of Category A or B to That of All Leaves (%) *^1^								Percentage of the Number of Category A or B to That of All Stems (%) *^2^							
	A *^3^				B *^4^				A *^3^				B *^4^			
	>95				>75				>95				>75			
	Focal Length of Lens (mm)															
	24	28	35	50	24	28	35	50	24	28	35	50	24	28	35	50
1	34	47	31	32	57	76	74	62	0	0	0	0	11	11	11	0
2	20	27	27	22	54	59	64	68	44	57	44	33	67	78	78	67
1 + 2	39	45	28	22	59	68	66	64	56	78	44	33	67	78	56	56
3	3	5	1	0	11	24	9	5	78	100	100	33	89	100	100	44
1 + 2 + 3	9	26	27	0	49	50	69	0	78	89	33	0	89	89	56	0
4	4	5	4	3	14	19	22	5	44	56	44	22	56	56	78	44
1 + 2 + 3 + 4	7	14	9	16	42	46	41	68	89	89	22	33	89	100	44	44

*^1^ The total number of real plant leaves is 74; *^2^ The total number of real plant stems is 9; *^3^ Category A means the percentage of 3D model shape surface area reconstructed over 95% compared to the real shape; *^4^ Category B means the percentage of 3D model shape surface area reconstructed over 75% compared to the real shape.

**Table 3:** The error results of the 3D model of the best leaf and stem taken by the 28-mm lens.

Camera Positions	Leaf		Stem	
	R^2^	RMSE (mm)	R^2^	RMSE (mm)
1	0.9821	1.65		
3			0.9998	0.57

## Abbreviations

The following abbreviations are used in this manuscript:

MCP	Multi-Camera Photography
MVS	Multiple-View Stereovision
SfM	Structure from Motion
RMSE	Root-Mean-Square Error
SIFT	Scale-Invariant Feature Transform
PPF	Photosynthesis Photon Flux
PnP	Perspective N-Point
SSD	Sum of Squared Differences
DEM	Digital Elevation Model
